# The South Shields Sanatorium

**Published:** 1920-11-13

**Authors:** 


### The South Shields Sanatorium.

The tender of Mr. A. Pringle, Gateshead, for a sanatorium of 100 beds and an infectious diseases hospital of eighty-eight beds, to be built on the Cleadon Park Estate, South Shields, has been accepted for £141,079. With their equipment the new institutions are expected to cost £145,000. A grant of £180 per bed in respect of the sanatorium will be made by the Ministry of Health if the work is carried out within two and a-half years of the date of the Ministry's approval. An annual grant will be made of half the net deficit on the maintenance account of the sanatorium.

